# Clinical Audit of the Incidence and Documentation Quality of Contrast-Induced Nephropathy (CIN) in a Tertiary Cardiology Unit

**DOI:** 10.7759/cureus.112270

**Published:** 2026-07-08

**Authors:** Aleena Sunny, Ajith Chandran, Hashir Kareem

**Affiliations:** 1 Medicine, KIMSHEALTH, Trivandrum, IND; 2 Cardiology, KIMSHEALTH, Trivandrum, IND

**Keywords:** acute kidney injury, clinical audit, contrast-induced nephropathy (cin), contrast-media, renal function documentation, serum creatinine monitoring

## Abstract

Introduction: Contrast-induced nephropathy (CIN) is a potentially preventable form of acute kidney injury triggered by intravascular iodinated contrast, with risk reduction achievable through hydration and appropriate patient selection. Reliable diagnosis requires accurate pre- and post-procedure serum creatinine monitoring. Missing creatinine documentation can significantly impair the detection of true CIN incidence.

Aim: To assess CIN incidence, evaluate adherence to renal protection protocols, and quantify missing creatinine documentation among patients undergoing contrast procedures.

Methods: A retrospective clinical audit was conducted from January to August 2025, involving 172 patients undergoing contrast-based cardiology procedures. Patients were divided equally into Cycle 1 (January-April 2025, n=86) and Cycle 2 (May-August 2025, n=86). Cycle 1 included patients with both complete and incomplete creatinine documentation, while Cycle 2 comprised exclusively fully documented patients. CIN was defined per the European Society of Urogenital Radiology (ESUR) criteria as an absolute rise in serum creatinine of ≥0.5 mg/dL or a relative rise of ≥25% from baseline within 48-72 hours, or per the Kidney Disease: Improving Global Outcomes (KDIGO) criteria as ≥0.3 mg/dL or ≥50% rise.

Results: Among the 172 patients, 39 (22.7%) had at least one missing creatinine measurement. Specifically, 4 patients (2.3%) lacked pre-procedure values, 37 (21.5%) lacked post-procedure values, and 2 (1.1%) lacked both (accounted for in both categories). The two patients missing both measurements are included in both the pre- and post-procedure missing categories. All 39 patients with missing documentation belonged to Cycle 1. Cycle 2 demonstrated 100% compliance with creatinine documentation. Due to missing post-procedure data, CIN incidence could not be fully assessed in Cycle 1. No CIN cases were identified in Cycle 2 among patients with complete data.

Conclusion: The audit revealed that missing post-procedure creatinine documentation was the primary barrier to accurate CIN detection. Targeted interventions, including mandatory creatinine order sets, electronic medical record (EMR) prompts, and checklist integration, are recommended. Documentation improved in Cycle 2, supporting the benefit of workflow reinforcement. A re-audit is planned following the implementation of corrective measures.

## Introduction

Contrast-induced nephropathy (CIN) is a recognized complication following exposure to intravascular iodinated contrast during cardiac and radiological procedures [1,2]. CIN represents one of the leading causes of hospital-acquired acute kidney injury (AKI) and is associated with prolonged hospitalization, adverse cardiovascular outcomes, and increased mortality [3,4]. The Kidney Disease: Improving Global Outcomes (KDIGO) criteria define CIN as an acute rise in serum creatinine of ≥0.3 mg/dL or ≥50% above baseline within 48-72 hours of contrast exposure [5]. The European Society of Urogenital Radiology (ESUR) uses a threshold of ≥0.5 mg/dL or ≥25% rise [6]. More recently, the updated European Society of Urogenital Radiology (ESUR) recommendations by van der Molen et al. further refined the terminology, advocating for the term 'post-contrast AKI' (PC-AKI) to reflect the multifactorial nature of renal impairment following contrast exposure [7,8]. For consistency with historical audit standards and existing literature, this audit retains the term CIN. Patients with chronic kidney disease (CKD), diabetes mellitus, dehydration, advanced age, hypotension, and higher contrast volumes are at increased risk [1,2,9].

Accurate monitoring of renal function through timely pre- and post-procedure serum creatinine testing is fundamental to CIN detection and prevention [5,10]. The Mehran risk score, widely used in clinical practice, integrates patient- and procedure-related variables to stratify CIN risk following percutaneous coronary intervention [10]. However, CIN remains underreported and frequently overlooked due to inconsistent documentation of renal function tests, particularly post-procedure creatinine levels [11,12]. The American College of Radiology-National Kidney Foundation (ACR-NKF) consensus statements have similarly emphasized that the perceived nephrotoxic risk of contrast media has been historically overstated, partly due to the absence of adequate control groups and inconsistent follow-up documentation [13]. In many healthcare settings, including cardiology units, gaps in documentation prevent reliable assessment of CIN incidence and hinder implementation of preventive measures such as hydration protocols and renal function monitoring [3,6].

This clinical audit was undertaken to evaluate adherence to renal protection protocols among patients undergoing contrast-based cardiology procedures in a tertiary cardiac unit. In addition to assessing CIN incidence, the audit introduced a new objective: quantifying the extent and impact of missing creatinine documentation. This is essential for establishing accurate CIN surveillance, identifying workflow gaps, and implementing appropriate quality improvement interventions.

## Materials and methods

Study design, setting, and duration

This retrospective clinical audit was conducted in the cardiology department of a tertiary care teaching hospital over eight months (January-August, 2025). The audit was structured using the clinical audit cycle framework, which involves establishing standards of care, measuring current practice against those standards, identifying gaps, implementing changes, and re-auditing to assess improvement [11]. The primary aim was to evaluate renal function monitoring practices among patients undergoing contrast-enhanced cardiac procedures, including diagnostic coronary angiography and percutaneous coronary interventions (PCI). The secondary aim was to quantify the extent and impact of missing serum creatinine documentation on the ability to assess CIN incidence.

The audit was divided into two cycles of equal duration and sample size to allow comparison of workflow adherence before and after internal process reinforcement. Cycle 1 (January-April 2025) served as the baseline assessment period, during which standard clinical practices were followed without any specific interventions or reminders regarding creatinine documentation. Following analysis of Cycle 1 findings, internal process reinforcement measures were communicated to the clinical team, including verbal reminders to cardiology fellows, nursing staff, and laboratory personnel regarding the importance of documenting both pre- and post-procedure creatinine values. Cycle 2 (May-August 2025) was conducted to evaluate whether these informal interventions improved documentation compliance and enabled accurate CIN assessment.

Audit standards

The audit standards were derived from the KDIGO Clinical Practice Guideline for Acute Kidney Injury (2012) [5] and the ESUR Contrast Media Safety Guidelines (version 10.0, 2018) [6]. Additional guidance was drawn from the updated ESUR PC-AKI recommendations [7,8] and the ACR-NKF consensus statements [13]. It is acknowledged that the KDIGO 2026 Clinical Practice Guideline for Acute Kidney Injury (AKI) and Acute Kidney Disease (AKD), which represents the first major update since 2012, is currently available as a public review draft; however, as it had not been formally published at the time this audit was conducted, the 2012 guideline was retained as the reference standard. The following six standards were established as benchmarks against which clinical practice was measured, as presented in Table 1. Further, a corresponding pre- and post-contrast renal monitoring checklist was developed to operationalize these standards at the bedside (Table 2).

**Table 1:** Renal protection standards adapted from KDIGO/ESUR Audit standards were derived from the Kidney Disease: Improving Global Outcomes (KDIGO) Clinical Practice Guideline for Acute Kidney Injury [5] and the European Society of Urogenital Radiology (ESUR) Contrast Media Safety Guidelines (version 10.0, 2018) [6]. CIN is defined as an acute rise in serum creatinine of ≥0.3 mg/dL or ≥25% above baseline within 48-72 hours of contrast exposure per the KDIGO criteria [5]. Hydration recommendations are based on ESUR guidelines advocating IV isotonic saline administration to reduce CIN risk [6]. The 100% compliance target for each standard was established by the audit team based on best practice recommendations from these guidelines. CIN: Contrast-Induced Nephropathy; eGFR: estimated Glomerular Filtration Rate; KDIGO: Kidney Disease: Improving Global Outcomes; ESUR: European Society of Urogenital Radiology; RFT: Renal Function Test

S. No	Standard	Required Compliance	Description
1	Pre-procedure serum creatinine (urea, creatinine, eGFR, electrolytes) within 48 hours	100%	Baseline renal function must be documented for all patients before receiving iodinated contrast.
2	Adequate hydration before contrast exposure	100%	Hydration via oral fluids or IV isotonic saline as per KDIGO/ESUR recommendations to reduce CIN risk.
3	Post-procedure serum creatinine within 48-72 hours	100%	Essential to detect CIN (≥0.3 mg/dL or ≥25% rise in creatinine from baseline).
4	Post-procedure hydration	100%	Continued hydration after contrast administration to optimize renal perfusion.
5	Documentation of both pre- and post-creatinine, enabling CIN evaluation	100%	Complete renal monitoring required to calculate true CIN incidence.
6	Prompt nephrology consultation for deranged post- contrast renal function	100%	Patients meeting CIN criteria should be referred to nephrology for further evaluation.

**Table 2:** Pre- and post-contrast checklist used for audit comparison This checklist was developed by the authors for the purpose of this clinical audit, based on recommendations adapted from the KDIGO Clinical Practice Guideline for Acute Kidney Injury [5], the ESUR Contrast Media Safety Guidelines [6], and the Mehran risk stratification model for contrast-induced nephropathy [10]. Pre-contrast requirements ensure baseline documentation and prophylactic hydration, while post-contrast requirements facilitate timely CIN detection and nephrology referral. The CIN risk stratification criteria (item 5) were informed by established risk factors identified by Seeliger et al. [1], McCullough et al. [2], and Sudarsky et al. [9]. CIN: Contrast-Induced Nephropathy; CKD: Chronic Kidney Disease; eGFR: estimated Glomerular Filtration Rate; RFT: Renal Function Test; KDIGO: Kidney Disease: Improving Global Outcomes; ESUR: European Society of Urogenital Radiology

S. No	Pre-contrast Requirements	Post-contrast Requirements
1	Are baseline renal function tests (urea, creatinine, eGFR, electrolytes) documented?	Repeat RFTs (urea, creatinine, eGFR, electrolytes) within 48-72 hours after contrast exposure.
2	If inpatient: has the creatinine been checked within 48 hours before the procedure?	If inpatient, ensure post-procedure creatinine is done before discharge.
3	If outpatient, is the patient instructed to undergo baseline RFTs before the procedure?	If outpatient, the patient must return after 48 hours for repeat RFTs.
4	Was hydration administered (oral/IV) before the contrast exposure?	Hydration advised and monitored post-procedure.
5	Is CIN risk-stratified? (CKD, diabetes, elderly >70, hypotensive, high contrast volume)	If creatinine rise ≥25% or ≥0.3 mg/dL, notify the nephrology team.

Sample size and selection

A total of 172 unique patient records were identified during the study period through a systematic review of the cath-lab procedural logbook and cross-referenced with the hospital's electronic medical record (EMR) system. After data cleaning, patients were divided evenly into two cycles: Cycle 1 (n=86), which included 39 patients with missing creatinine documentation and 47 fully documented cases, and Cycle 2 (n=86), in which all patients had complete creatinine documentation.

Inclusion criteria

The following inclusion criteria were applied: (a) patients aged greater than 18 years undergoing contrast-based cardiology procedures, including diagnostic coronary angiography, PCI, or peripheral vascular angiography; and (b) availability of hospital-generated laboratory and procedure records within the institutional EMR system.

Exclusion criteria

The following patients were excluded from the audit: (a) patients with incomplete identification data that precluded accurate record linkage; (b) patients undergoing non-contrast cardiac procedures, such as echocardiography or electrophysiology studies; (c) patients with pre-existing CKD stages 4-5 (eGFR <30 mL/min/1.73 m²), as these patients follow distinct renal monitoring protocols; (d) patients who received additional intravenous contrast (e.g., CT scans) within 48 hours before or after the cardiac procedure, as this would confound CIN attribution; (e) patients whose laboratory investigations were performed at external facilities and whose results were not available within the hospital records; and (f) patients undergoing emergency procedures where standard hydration protocols were not feasible due to clinical urgency.

Data collection

Data were extracted retrospectively from multiple institutional sources by the primary investigator (AS) using a structured data collection proforma designed specifically for this audit. The data sources included EMR, the laboratory information systems (LIS), cath-lab procedure reports, and medication and hydration charts maintained by nursing staff.

The following variables were collected for each patient: demographic data (age and gender), type of cardiac procedure performed (diagnostic coronary angiography, PCI, or peripheral angiography), pre-procedure serum creatinine value (documented within 48 hours prior to contrast exposure), post-procedure serum creatinine value (documented within 48-72 hours after contrast exposure), presence or absence of creatinine documentation at each time point, CIN status based on KDIGO criteria (≥0.3 mg/dL absolute rise or ≥25% relative rise from baseline), hydration status where documented (type and volume of IV fluids administered), and categorical flags for missing creatinine values.

All missing values were identified and categorized into three groups: (a) missing pre-procedure creatinine only, (b) missing post-procedure creatinine only, and (c) missing both pre- and post-procedure creatinine. For patients with missing creatinine, alternative indicators of AKI (e.g., urine output, clinical signs) were not systematically recorded. This categorization was essential for determining the proportion of patients in whom CIN incidence could be accurately assessed.

Data analysis

Data were entered into Microsoft Excel (version 2021; Microsoft Corporation, Redmond, WA) and analysed using descriptive statistics. Continuous variables (age, creatinine values) were summarized as mean ± standard deviation (SD) or median with interquartile range (IQR) as appropriate. Categorical variables (gender, procedure type, documentation completeness, CIN status) were presented as frequencies and percentages. Completeness of renal monitoring documentation was quantified for each cycle and compared between Cycle 1 and Cycle 2. Missing documentation patterns were quantified. CIN incidence could only be evaluated in fully documented patients. Comparative bar graphs were generated to visually demonstrate adherence gaps, missing documentation proportions, and cycle-based improvements.

Ethical considerations

Formal ethics committee approval was not required, as this was a retrospective clinical audit of anonymized patient records conducted as part of routine quality improvement processes within the institution, consistent with established guidelines for clinical audit methodology [14]. Authorization for data access and audit conduct was obtained from the medical director of the cardiology unit. All patient data were de-identified prior to analysis, and no patient interventions were conducted outside standard clinical care. The audit was conducted in compliance with the principles of the Declaration of Helsinki.

## Results

Demographic characteristics

A total of 172 patients undergoing contrast-based cardiology procedures were included in this audit, with 86 patients in each cycle. The demographic characteristics of the study population are presented in Table 3.

**Table 3:** Demographic characteristics of the study population PCI: Percutaneous Coronary Intervention; SD: Standard Deviation

Characteristic	Cycle 1 (n=86)	Cycle 2 (n=86)	Total (N=172)
Age (years), Mean ± SD	58.4 ± 11.2	59.1 ± 10.8	58.7 ± 11.0
Male, n (%)	62 (72.1%)	64 (74.4%)	126 (73.3%)
Female, n (%)	24 (27.9%)	22 (25.6%)	46 (26.7%)
Coronary Angiography, n (%)	52 (60.5%)	50 (58.1%)	102 (59.3%)
PCI, n (%)	30 (34.9%)	32 (37.2%)	62 (36.0%)
Peripheral Angiography, n (%)	4 (4.6%)	4 (4.7%)	8 (4.7%)

Missing creatinine documentation

**Figure 1:**
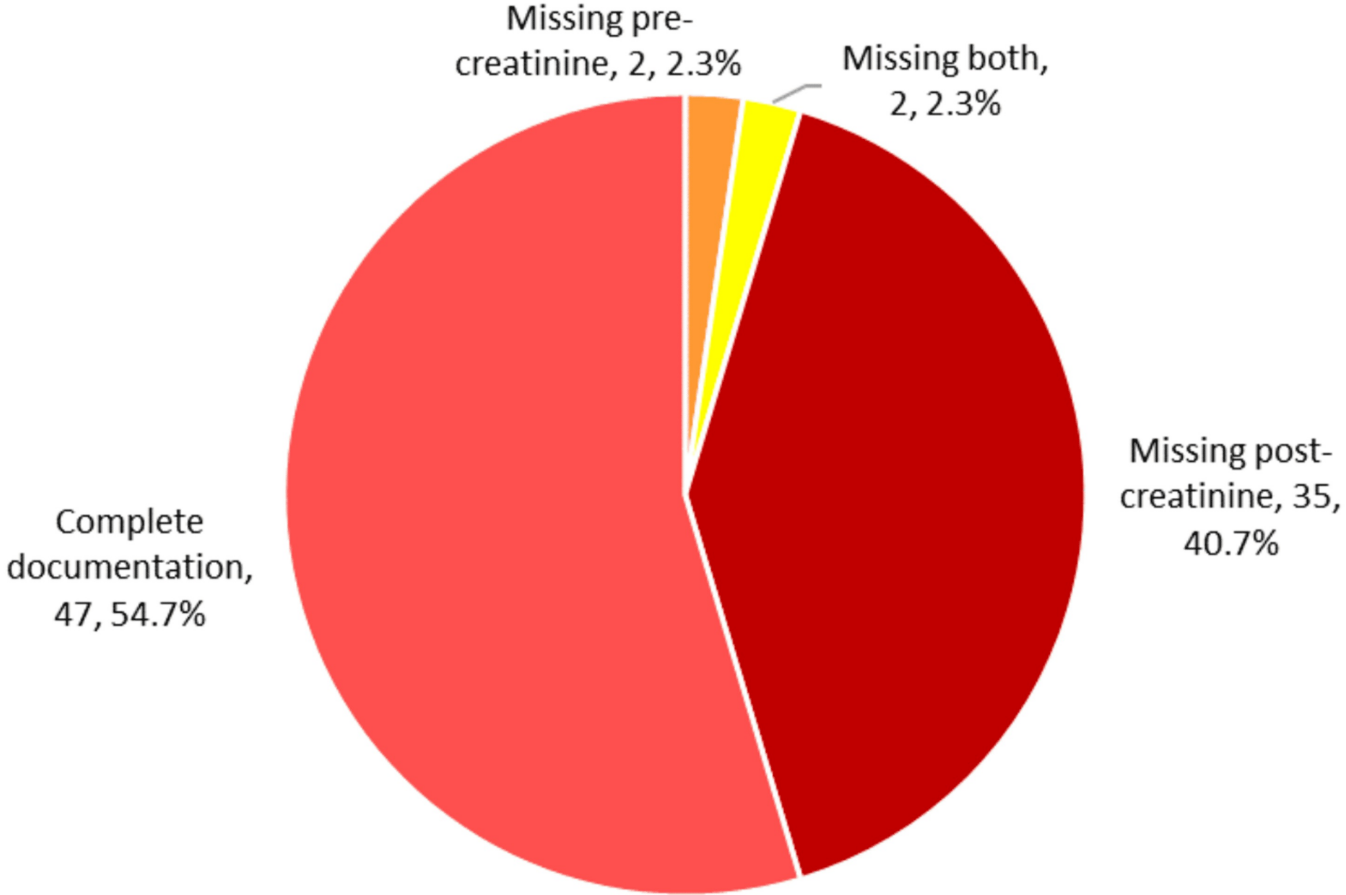
Proportion of missing creatinine documentation in Cycle 1

The overall rate of missing creatinine documentation was substantial. Among all 172 patients, 39 (22.7%) had at least one missing creatinine value. When classified into mutually exclusive categories, in Cycle 1, 2 patients (2.3%) had missing pre-procedure creatinine only, 35 (40.7%) had missing post-procedure creatinine only, and 2 (2.3%) had both values missing. In total, four patients (4.7%) lacked any pre-procedure creatinine, and 37 (43.0%) lacked any post-procedure creatinine, yielding 39 unique patients. All cases of missing documentation occurred exclusively in Cycle 1 (Figure 1). Cycle 2 had complete documentation for all 86 patients (100%).

Cycle 1 findings

Cycle 1 demonstrated the greatest deficiency in documentation. Out of 86 patients, 39 had at least one missing creatinine value, primarily due to absent post-procedure renal function testing. While 82 of 86 patients (95.3%) had pre-procedure creatinine recorded, only 49 of 86 patients (57.0%) had post-contrast creatinine collected within the recommended 48-72-hour window, resulting in only 47 of 86 patients (54.7%) having complete pre- and post-procedure documentation. The high proportion of missing data in this cycle highlighted poor adherence to recommended renal monitoring protocols and precluded accurate assessment of CIN incidence for a substantial proportion of patients (Table 4).

**Table 4:** Comparison of renal protection standards and actual adherence during both audit cycles Cycle 1 showed a major deficit in post-procedure creatinine documentation, while Cycle 2 achieved complete compliance with monitoring recommendations.

Standard (KDIGO/ESUR)	Required	Cycle 1 (n=86)	Cycle 2 (n=86)
Pre-procedure creatinine within 48 hours	100%	82/86 (95.3%)	86/86 (100%)
Post-procedure creatinine within 48-72 hours	100%	49/86 (57.0%)	86/86 (100%)
Complete creatinine documentation (pre + post)	100%	47/86 (54.7%)	86/86 (100%)
Ability to assess CIN incidence	100%	Not possible (missing post-creatinine)	Fully possible
Optimal/Full adherence to CIN protocol	100%	Suboptimal	Compliant

Cycle 2 findings

Cycle 2 showed marked improvement in documentation completeness. All 86 patients in this cycle had both pre- and post-procedure creatinine values recorded, achieving 100% compliance across all documentation standards. This enabled a complete CIN assessment for all patients in this cycle. No cases of CIN (as defined by the ESUR criteria: ≥0.5 mg/dL or ≥25% increase within 48-72 hours) were identified among fully documented patients in Cycle 2.

Comparison between cycles

When comparing both cycles, Cycle 1 had substantially higher rates of missing creatinine documentation, whereas Cycle 2 achieved full adherence to renal monitoring guidelines. This improvement reflects enhanced workflow consistency and increased awareness regarding the importance of routine post-contrast renal function assessment. A summary of compliance with audit standards across both cycles is presented in Table 4.

Impact on CIN evaluation

Due to incomplete documentation in Cycle 1, CIN incidence could not be reliably assessed for that cycle. Conversely, Cycle 2 provided complete data, demonstrating zero CIN cases among fully documented patients. This emphasizes that missing data, not true CIN incidence, was the main barrier to accurate CIN assessment.

Summary

**Figure 2:**
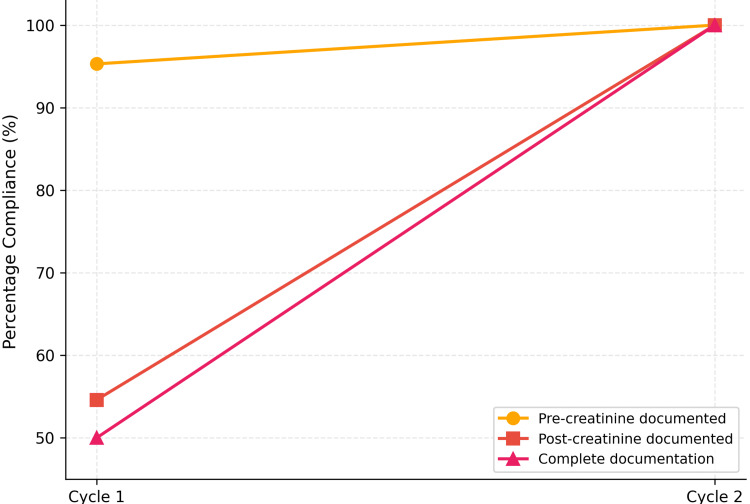
Improvement trend of documentation between cycles

Overall, the audit revealed significant gaps in renal monitoring protocols during Cycle 1, mainly attributed to missing post-procedure creatinine. Full compliance achieved in Cycle 2 suggests improvement in team awareness and adherence to guidelines. The improvement trend across cycles is depicted in Figure 2. These findings underscore the need for system-level interventions to ensure consistent and reliable CIN monitoring.

## Discussion

This clinical audit assessed adherence to renal monitoring standards for detecting CIN in a tertiary cardiology unit. The most significant finding was the substantial amount of missing serum creatinine documentation, particularly in Cycle 1, which prevented accurate assessment of CIN incidence. The observed deficiencies in Cycle 1 were attributable to incomplete post-contrast renal function documentation rather than confirmed changes in renal function [1,3].

Incomplete documentation is a recognized obstacle to CIN surveillance and quality improvement. In Cycle 1, although most patients (95.3%) had pre-procedure creatinine documented, 43% lacked post-procedure values. The mortality burden associated with renal dysfunction, accounting for over 25% of sudden adult deaths, underscores the importance of systematic post-contrast creatinine monitoring as a quality indicator in cardiology units [15]. This mirrors patterns seen in other audits, where baseline renal assessment is often completed, but post-contrast monitoring is neglected due to workflow gaps, lack of automated reminders, or poor communication between departments [9,11,12]. The updated ESUR guidelines by van der Molen et al. similarly emphasized the critical importance of systematic post-contrast creatinine assessment, noting that the risk of post-contrast AKI has been historically overestimated partly due to absent control groups and inconsistent documentation practices [7,8]. Missing post-procedure creatinine leads to underestimation of CIN, incomplete risk stratification, and missed opportunities for timely hydration or nephrology referral [2,9,10]. McCullough et al. emphasized that CIN is the third most common cause of hospital-acquired AKI, further underscoring the importance of systematic screening and documentation [3]. This finding is further supported by the ACR-NKF consensus statements, which highlighted that inadequate documentation of post-contrast renal function contributes to both overestimation and underestimation of true contrast-associated kidney injury incidence [13].

The marked improvement seen in Cycle 2 - with 100% documentation of both pre- and post-procedure creatinine (Figure 1) - suggests that awareness, reinforcement of workflow expectations, and internal communication may correct deficiencies even without major systemic interventions [10]. Full compliance in Cycle 2 allowed for accurate CIN assessment, and no CIN cases were identified, supporting the assumption that missing documentation rather than true renal impairment was the primary barrier to CIN detection. Similar findings have been reported by Khandy et al., who demonstrated that structured monitoring protocols in catheterization laboratories were associated with improved CIN detection rates and more reliable risk stratification [16].

Our findings reinforce the need for standardized CIN monitoring protocols, including automated post-procedure creatinine orders, integrated checklists, and mandatory documentation prompts within the EMR. Similar interventions have been shown in other institutions to significantly improve adherence and reduce missed renal function assessments [3,11,16]. Implementing these measures would allow consistent CIN surveillance, early identification of at-risk patients, and targeted preventive strategies such as hydration therapy [1,2,9]. James et al. demonstrated in a systematic review that CI-AKI is consistently associated with mortality, cardiovascular events, and prolonged hospital stay, reinforcing the clinical imperative of accurate documentation and early detection [4].

Recommendations

Based on the findings of this clinical audit, several evidence-based recommendations are proposed to improve adherence to renal monitoring protocols and ensure accurate detection of CIN. These recommendations are organized into system-level interventions, workflow modifications, and educational strategies, and are discussed in detail below.

First, the implementation of mandatory pre- and post-procedure creatinine order sets within the EMR is strongly recommended, though institutions should monitor for alert fatigue and avoid over-testing in low-risk patients. Integrating automatic creatinine orders for all contrast-based procedures will substantially reduce the likelihood of missed laboratory tests and ensure timely monitoring of renal function. This approach leverages the existing EMR infrastructure to create a default standard of care, removing reliance on individual physician memory or manual order entry. Studies have demonstrated that computerized physician order entry systems with embedded clinical decision support significantly improve adherence to laboratory monitoring protocols [3,10,13,16,17].

Second, the introduction of automated alerts and reminder systems for post-contrast creatinine testing at 48-72 hours, directed to the primary nursing team for inpatients and to the patient via SMS or phone for outpatients, is recommended. A scheduled notification system targeting nursing staff and treating physicians will help ensure that both inpatients and outpatients complete post-procedure renal testing within the recommended timeframe. For inpatients, this would involve an automated flag in the nursing task list, while for outpatients, SMS or telephone reminders could be employed to prompt patients to return for follow-up blood testing [1,5,6]. Recent evidence supports that structured post-procedure surveillance, combined with automated alerts, can reduce the gap between recommended and actual monitoring practices, particularly in high-volume catheterization laboratories [7,8,15].

Third, the incorporation of a comprehensive renal monitoring checklist into the cath-lab and radiology workflow is proposed. This checklist should document the following at each stage: pre-procedure creatinine value, hydration status (type, volume, and duration of IV fluids), post-procedure creatinine order placement, CIN risk factor assessment (including diabetes, CKD status, age, contrast volume), and follow-up plan. The use of standardized checklists has been shown to reduce errors and improve compliance in procedural settings [11,14].

Fourth, strengthening interdepartmental communication between cardiology, nephrology, radiology, and laboratory medicine is essential. The audit findings indicate that a significant proportion of missing documentation may stem from communication gaps between the procedural team and the laboratory. Establishing a formal liaison process or designating a CIN coordinator within the cardiology unit could improve follow-up and ensure that creatinine samples are collected and processed in a timely manner.

Fifth, regular staff education and refresher training sessions should be conducted to emphasize CIN risk factors, KDIGO diagnostic criteria, and the clinical consequences of missed documentation. Training should target all cadres of healthcare professionals involved in contrast procedures, including cardiologists, interventional fellows, nursing staff, and laboratory technicians [5,7,16,18].

Sixth, a structured outpatient follow-up process should be established for patients who undergo same-day contrast procedures and are discharged within 24 hours. These patients should receive clear written instructions and automated reminders to return at 48-72 hours for repeat creatinine testing. This is particularly important given that a significant proportion of contrast procedures are performed on an outpatient basis, and these patients are at the highest risk of having their post-procedure creatinine missed.

Seventh, monitoring compliance through continuous quality improvement (CQI) cycles is recommended. Regular internal audits at quarterly intervals will help maintain adherence, identify emerging workflow problems, and track the effectiveness of implemented interventions [11,14]. Finally, these monitoring standards should be expanded hospital-wide to include radiology, emergency, and intensive care departments where iodinated contrast media are routinely used [3,6].

Limitations

This audit has several limitations. First, missing creatinine documentation, especially post-procedure values, restricted the ability to assess CIN incidence in Cycle 1 [5,11]. Second, the audit relied solely on hospital records; external laboratory results or outpatient follow-up creatinine values were not captured, which may have led to underreporting. Third, the audit was conducted within a single tertiary cardiology unit, which may limit generalizability to other settings with different workflows or patient populations. Fourth, as demographic data, including specific comorbidities (diabetes, hypertension, and baseline eGFR for patients with stage 3 CKD), were not systematically recorded in Cycle 1, a detailed risk factor analysis could not be performed.

Finally, as this was a retrospective audit, causation cannot be inferred, and improvements in Cycle 2 may reflect increased awareness and the Hawthorne effect rather than formal interventions [10,16]. Despite these limitations, the audit provides valuable insight into renal documentation practices and highlights key areas for protocol improvement.

## Conclusions

This clinical audit identified significant gaps in renal monitoring practices, with missing post-procedure creatinine documentation being the key barrier to accurate detection of CIM. While Cycle 1 demonstrated poor adherence to recommended guidelines, Cycle 2 achieved full compliance, enabling a complete assessment of CIN incidence. The improvement between cycles highlights the effectiveness of workflow reinforcement and the potential for sustained enhancement through system-level interventions.

To ensure reliable CIN surveillance, this audit supports implementing mandatory creatinine order sets, automated reminders at 48-72 hours, and checklist-based monitoring. A re-audit following implementation of these interventions is planned to confirm sustained adherence and further strengthen patient safety in the setting of contrast exposure.
